# Ectopic third molar in the mandibular sigmoid notch: Report of a case and literature review

**DOI:** 10.4317/jced.51871

**Published:** 2015-02-01

**Authors:** Yavuz Fındık, Timuçin Baykul

**Affiliations:** 1 DDS, PhD, Assistant Professor, Department of Oral and Maxillofacial Surgery, Faculty of Dentistry, Süleyman Demirel University, Isparta, Turkey; 2 DDS, PhD, Professor, Department of Oral and Maxillofacial Surgery, Faculty of Dentistry, Süleyman Demirel University, Isparta, Turkey

## Abstract

Purpose: To evaluate the etiopathogenesis, clinical features and surgical approaches for removal of ectopic third molars in the mandible. Methods: We report a case of an impacted mandibular third molar dislocated on mandibular sigmoid notch. 20 cases of ectopic mandibular third molars reported in the English-language literature, identified from Pubmed and Medline databases are also reviewed. Results: Among the 20 article reported in the presented study, ectopic third molars were generally located in the condylar region. The common symptoms of the clinical examination were pain, trismus, swelling, temporomandibular joint syndroms or no symptoms. Conclusions: Ectopic third molar may be asymptomatic initially with clinical manifestations, later on as adjacent structures are affected. The surgical approach must be carefully planned for the aim of choosing the more conservative technique that produces the minimum trauma to patients.

** Key words:**Ectopic third molar, sigmoid notch, cyst.

## Introduction

Ectopic teeth often impact in unusual positions or at a distance from their normal anatomic location. Ectopic eruption of a tooth into dental structures is a common entity, while ectopic eruption of a tooth in other sites is infrequent (1). Reported sites include the maxillary sinus, palate, mandibular condyle, coronoid process, orbit, nasal cavity or through the skin (2,3). Ectopic teeth may be supernumerary, deciduous, or permanent.

Permanent mandibular third-molar impaction is a common condition in populations, with a frequency of 20% to 30%. Ectopic mandibular third molars, however, are unusual, with their heterotopic positions reported in the condylar area, in the ascending ramus of the mandible, or in the coronoid process. Most cases of ectopic third molars are asymptomatic and are usually found during routine clinical and radiographic investigations. In the present study, we report a case of an ectopic third molar on the right sigmoid notch with multiple cysts and compare it with the literature reports of this anomaly.

## Material and Methods

We performed a literature search using the PubMed database. We used ectopic third molar and mandible as key words. Of the 45 articles found, we only considered the English literature and well-documented cases of ectopic third molars in the mandible. Our inclusion criteria were as follows: English-language articles generated by the Pubmed database search containing at least one case of an ectopic third molar in the mandible; and articles containing cases of patients with ectopic third molars located in the mandibular condyle, coronoid, and sigmoid regions ( Table 1). We compared the results of our search with the following case study.

**Table 1:**
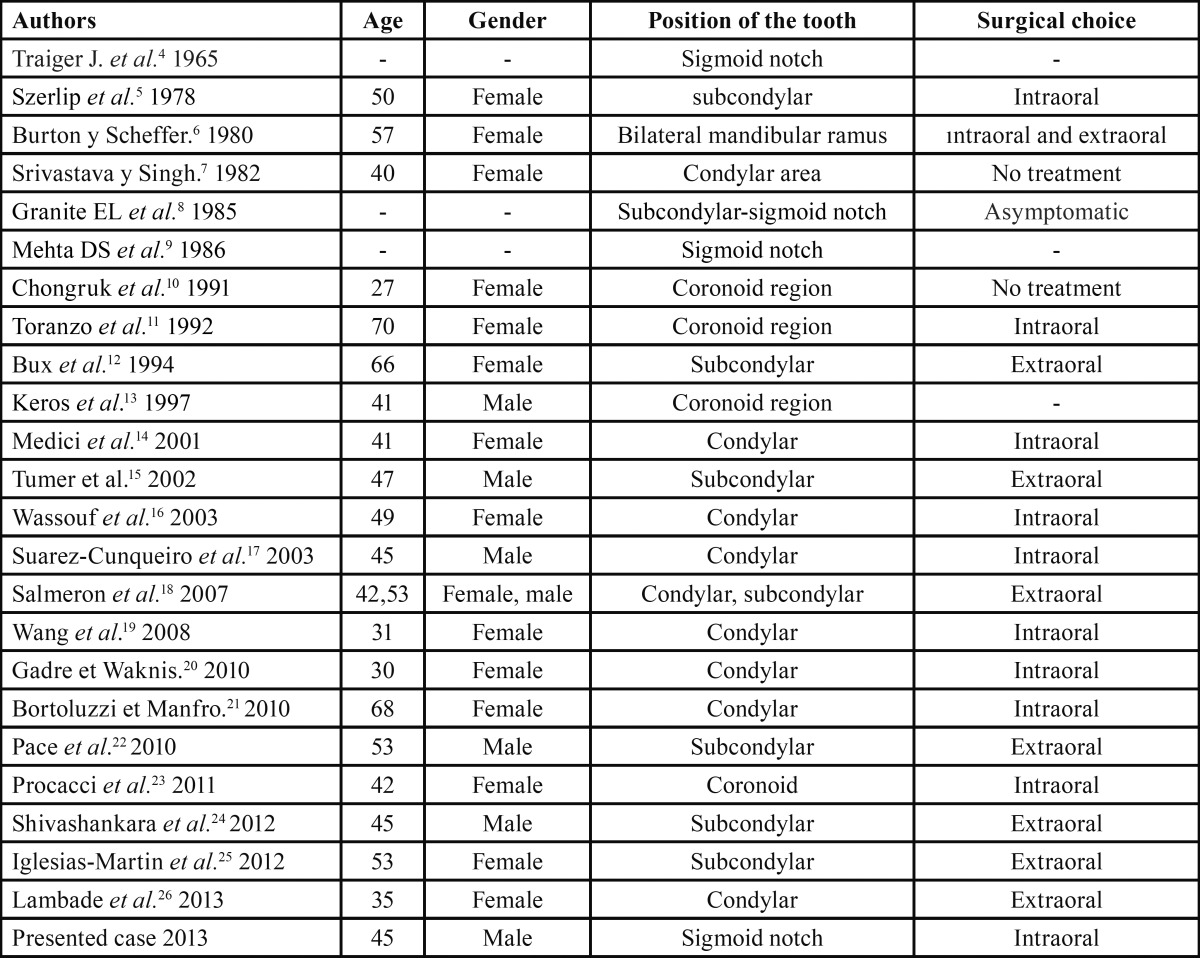
Ectopic third molars in the literature.

**Figure 1:**
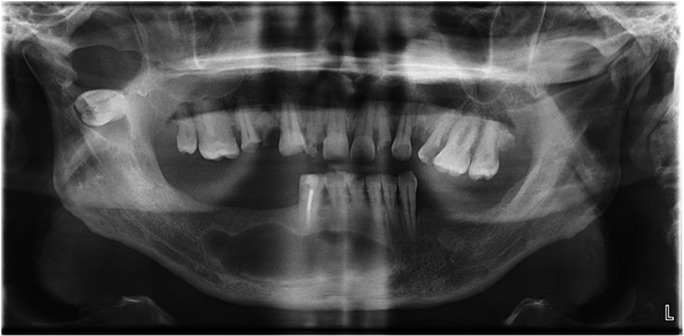
Panaromic view of the patient before the surgery.

**Figure 2:**
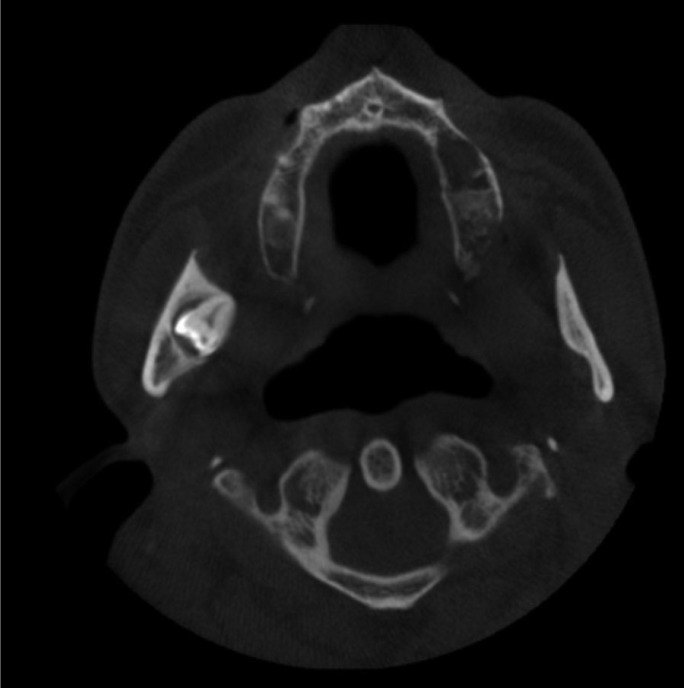
CBCT view of the ectopic third molar.

A 45-year-old man was referred to the Department of Oral and Maxillofacial Surgery, Faculty of Dentistry Hospital (Isparta, Turkey), in August 2011 for the evaluation and assessment of an impacted lower right third molar in the mandibular sigmoid notch associated with a radiolucent lesion and multiple cysts located in the jaws. His medical history also did not give any significant information that could associated with the presenting symptoms. On intra oral examination, there were no signs of any significant pathology in relation to any tooth or tissue. Panoramic radiograph examination revealed a radiolucent area surrounding the crown of the fully formed, ectopic mandibular third molar in right mandibular sigmoid notch region (Fig. 1). Cystic lesions were also seen in the left maxillary and right mandibular area. Cone beam CT scans showed the impacted tooth with the proximity to the lingual cortical bone and there was also cortical bone exposure in the coronal images (Fig. 2).

The surgical staff carried out the surgical procedure with the patient under general anesthesia. They obtained intraoral access via an incision on the anterior edge of the mandibular ramus and along the external oblique ridge. They performed the periosteal dissection was done lingually to expose the sigmoid notch of the mandible. They used a round burr was used to make cuts on the cortical bone of the mandible where they had estimated the crown of the ectopic tooth from the CT scans and the panoramic radiograph.

They elevated the tooth was then elevated out of the bony socket by an elevator via the bony window. Then they enucleated the other cystic lesions located in the maxilla and mandible, were enucleated without any complications. They sent the enucleated soft tissues were sent to pathology and sutured the wound after irrigation. The pathology report revealed tissue compatible with a dentigerous cyst around the ectopic tooth; radicular cysts in the maxilla; residual csyt in the mandible.

**Figure 3:**
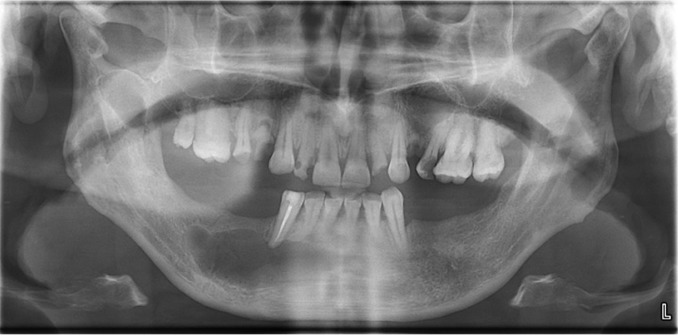
Panaromic view of the patient after the removal of the ectopic third molar and other cysts.

The postoperative phase was uneventful (Fig. 3). The patient was under antibiotic coverage along with anti-inflammatory analgesics for 7 days. The patient was under regular follow-up care for 18 months. There was no deviation of the mandible and preoperative occlusion was maintained without any functional discrepancy.

## Results

Among the 23 articles reported in the presented study, ectopic third molars were generally located in the condylar region. The common symptoms of the clinical examination were pain, trismus, swelling, temporomandibular joint syndrome or no symptoms (5-25). Fever with acute inflammation and drainage of purulent material through intraoral or extraoral areas have also occured (7,12). Among the 23 case reports summarized in the table, 7 were in men , 13 cases were in women and 3 were unknown.

Sixteen cases received extraction of the ectopic third molar. Ten of the ectopic third molars were extracted through intraoral access, and the other 6 were extracted through extraoral access. In the extraoral approaches, the most frequently used access routes were the submandibular and retromandibular, because they provide a good surgical exposure especially to the body and ascending ramus, and with a higher difficulty, to the condylar region. In one study, the authors presented an endoscopically assisted surgical technique for the removal of an ectopic lower third molar in the condylar process (17).

Generally, all studies described a radiolucent image around the ectopic molar upon radiography, with the diagnosis of a dentigerous cyst. The associated cyst was infected in some cases (6,11). The infected cyst in the coronoid process described by Toranzo *et al.* is the only case in which the adjacent cyst apparently did not encompass the crown of the ectopic third molar. In one case report, authors presented a case where an ectopically placed mandibular third molar led to an extraoral sinus and scar formation below the ear lobule with osteomyelitis of the mandibular condyle (26).

## Discussion

Ectopic teeth are located in the jawbones or regions other than the alveolar arch. Ectopic eruption of a tooth is rare; however, there have been few reports of tooth in the nose, mandibular condylary and coronoid processes and maxillary sinus (25,27,28). Most of the cases in the mandibular coronoid and condylary regions had symptomatic signs; common symptoms of the clinical examination were pain, trismus, swelling, and temporomandibular joint problems. On the other hand, ectopic teeths are often discovered in routine clinical or radiographic examinations; as some of the cases were asymptomatic, like the case study above.

The aetiology of ectopic eruption is still unclear, and reaches have suggested many theories, including trauma, infection, pathologic conditions, crowding and developmental anomalies. Odontogenesis is a complex process, and abnormal tissue interactions between the oral epithelium and the underlying mesenchymal tissue during development may potentially result in ectopic tooth development and eruption (29). A mandibular third molar may be displaced by a lesion such as a cyst or a tumor (14). The displacement of tooth buds by the expansion of progressively growing dentigerous cysts may result in the displacement of the tooth to other areas.

In some of the reports in the literature review, the cysts associated with the ectopic third molars were very small like our case. Such cysts may have once occupied the entire ramus, but their walls may have been perforated, which resulted in drainage and decompression (12,14). This pathological process may support the idea that a dentigerous cyst was the etiologic factor of ectopic eruption in the subject of our case study.

Most dentigerous cysts are solitary. Bilateral and multiple cysts are usually found in association with a number of syndromes that include cleidocranial dysplasia. In the absence of these syndromes, multiple cysts are rare.30 This is a report of the unusual occurrence of nonsyndromic adjacent cysts. A literature search has shown no report of a similar case.

Treatment of ectopic third molars in the coronoid and condylar regions is recommended to avoid the morbidity caused by infec-tion of the cyst, malfunction of the temporomandibular joint, and risk of fracture in an area with a very thin bone. In cases of symptom-free highly aberrant wisdom teeth or without urgent necessity, annual follow-up visits to monitor the growth of the lesion are appropriate.

In the cases described in the literature, surgeons have used several surgical approaches have been used, such as extraoral (preauricular, retromandibular, endoaural), intraoral, and recently, endoscopic approaches. The most commonly used extraoral approaches were submandibular access and preauricular approach. These external approaches have the advantage of good exposure of the surgical site but may result in complications such as extraoral scar formation, damage of joint components, facial nerve injury in the case of preauricular access, or damage of the marginal branch of the seventh cranial nerve in the case of submandibular access (14).

Surgeons have use endoscopy because it magnifies the surgical field. Especially in intraoral approaches, this is an important property. However, the cost of endoscopes and the lack of training facilities prohibit the routine use of endoscopy.

The selection of the surgical approach is basically linked to the experience and preference of surgeons. In one study, surgeons presented an endoscopically assisted surgical technique for the removal of an ectopic lower third molar in the condylar process associated with a dentigerous cyst (17). In a case report, surgeons used an endoaural approach that causes low morbidity from facial palsy and gives adequate exposure of the temporomandibular region (16). In the presented case, we used an intraoral approach to avoid visible scars and facial nerve injury. ın some cases, authors used miniplates, especially impacted third molars in the condyle, to avoid fractures (22) because they thought the loss of some bony support at the condylar neck post-surgery would increase the risk of a pathological fracture in such cases.

In our literature review, the authors agreed that the treatment should be carefully planned according to the location and position of the ectopic tooth and morbidity associated with surgery, with the aim of choosing the most conservative technique that produces the least possible trauma to the patient.

In conclusion, occurrence of an ectopic tooth in the coronoid or condylar regions and association of a dentigerous cyst with it is a rare phenomenon. Its presence may be the asymptomatic initially with later clinical manifestations as adjacent structures are affected. The surgical approach must be carefully planned for the aim of choosing the most conservative technique that produces the minimum trauma to patients. Postoperative follow-up with radiographic examination at regular intervals is mandatory to rule out any recurrence.
